# When the Mind Meets the Ear: A Scoping Review on Tinnitus and Clinically Measured Psychiatric Comorbidities

**DOI:** 10.3390/jcm14113785

**Published:** 2025-05-28

**Authors:** Virginie Arsenault, Jacob Larouche, Marie Désilets, Marc-Antoine Hudon, Alexandre Hudon

**Affiliations:** 1 Department of Surgery, Faculty of Medicine, Université de Montréal, Montreal, QC H3T 1J4, Canada; virginie.arsenault.med@ssss.gouv.qc.ca; 2 Department of Psychiatry and Addictology, Faculty of Medicine, Université de Montréal, Montreal, QC H3T 1J4, Canada; jacob.larouche@umontreal.ca; 3 Department of Psychiatry, Institut Universitaire en Santé Mentale de Montréal, Montreal, QC H1N 3M5, Canada; mdesilets.iusmm@ssss.gouv.qc.ca; 4 ENT Department, Hôpital Pierre-Bourcher, Longueuil, QC J4M 2A5, Canada; marc.antoine.hudon@usherbrooke.ca; 5 Faculty of Medicine, Université de Sherbrooke, Sherbrooke, QC J1N 3C6, Canada; 6 Centre de Recherche de l’Institut Universitaire en Santé Mentale de Montréal, Montreal, QC H1N 3V2, Canada; 7 Institut National de Psychiatrie Légale Philippe-Pinel, Montreal, QC H1C 1H1, Canada

**Keywords:** tinnitus, psychiatric comorbidity, mental health, depression, anxiety, psychosis, sleep disorders, psychological distress, cognitive behavioral therapy, scoping review

## Abstract

**Background/Objectives**:Tinnitus, the perception of sound without an external source, is a prevalent and often distressing condition with complex neurobiological and psychological underpinnings. A growing body of literature suggests a frequent co-occurrence between tinnitus and psychiatric symptoms such as anxiety, depression, and sleep disturbances. However, the extent to which these conditions are associated, and whether treatments targeting one domain impact the other, remains unclear. This scoping review aimed to (1) identify associations between tinnitus and mental health comorbidities, (2) evaluate whether tinnitus treatments affect psychiatric outcomes, and (3) explore whether psychiatric treatments influence tinnitus symptoms. **Methods**: A comprehensive search of PubMed, MEDLINE, Embase, PsycINFO, Cochrane Database of Systematic Reviews, and Google Scholar was conducted for articles published between January 2014 and May 2025. Eligible studies were written in English, French, or Spanish, focused primarily on tinnitus, included at least one co-occurring psychiatric condition, and described how tinnitus was evaluated. A total of 30 studies were included. Data were extracted and synthesized thematically. Study quality was assessed using the Mixed Methods Appraisal Tool and relevant Joanna Briggs Institute checklists. **Results**: Most studies reported significant associations between tinnitus and psychiatric symptoms, particularly anxiety, depression, stress, insomnia, and, in some cases, psychosis. Treatments aimed at tinnitus, such as eye movement desensitization and reprocessing and cognitive behavioral therapy, were sometimes associated with secondary improvements in mental health. Conversely, limited evidence suggested that psychiatric treatment, including antipsychotic medication and psychotherapy, may reduce tinnitus severity in selected cases. **Conclusions**: Tinnitus and psychiatric comorbidities frequently co-occur, and early evidence suggests that addressing one may benefit the other. Given the specific inclusion criteria, this review presents a selected subset of the broader literature, focusing only on studies that evaluated tinnitus alongside clinically measured psychiatric symptoms. Future research should prioritize integrated, longitudinal interventions to better understand these complex interactions.

## 1. Introduction

Tinnitus is defined as the perception of sound without an external auditory stimulus. Tinnitus, which is frequently characterized as ringing, buzzing, or hissing in the ears, affects 10% to 15% of adults globally, with 1% to 2% reporting severe, life-altering symptoms [1,2]. Several etiologies have been associated with tinnitus, such as exposure to noise, ototoxic drugs, ear infections, and age-related hearing loss [3]. In many instances, however, no discernible anatomical or auditory disease is identified [4]. More recent research indicates that tinnitus involves intricate central nervous system mechanisms, particularly within brain networks linked to emotion, attention, and memory, even though it was once thought to be a peripheral auditory disorder [5,6]. Due to these new findings, the correlation between tinnitus and mental health issues has gained more interest. Several studies have suggested that psychological elements, rather than just audiological ones, may be involved in tinnitus discomfort [7].

People with tinnitus have a high prevalence of psychiatric comorbidities, according to recent studies. Conditions such as depression, generalized anxiety disorder, post-traumatic stress disorder (PTSD), and sleeplessness have been consistently observed among tinnitus sufferers [8,9,10,11]. According to some estimates, up to 50% of those who have persistent tinnitus fit the criteria for at least one mental health condition [12]. The hypothesis that tinnitus and emotional dysregulation may have similar neurological underpinnings is supported by neuroimaging studies that show overlapping activation in brain regions such as the insula, amygdala, and anterior cingulate cortex [13]. Furthermore, psychological discomfort frequently contributes more to perceived disability than audiometric variables alone, thus exacerbating the subjective severity of tinnitus [14]. These results highlight the necessity of viewing tinnitus as a phenomenon with important psychological components in addition to a sensory ailment.

Despite these associations, the literature is still lacking in its examination of the long-term interactions between psychiatric disorders and tinnitus, especially the potential effects of treating one illness or the other. Although there is some evidence that psychiatric and psychotherapy therapies, including cognitive behavioral therapy (CBT), antidepressants, or eye movement desensitization and reprocessing (EMDR), help lessen the suffering associated with tinnitus, these results have not yet been consistently assessed [15,16,17]. On the other hand, not much research has looked at how tinnitus therapies like sound therapy, neuromodulation, or cochlear implants can help with co-occurring mental health issues. Clinicians are left with little advice on how to handle the reciprocal nature of this interaction in practice due to the lack of integrative, longitudinal, and intervention-focused investigations. Furthermore, cross-study comparisons are still hampered by differences in psychiatric outcome reporting and tinnitus evaluation techniques.

The aim of this scoping review is to map the body of research on the relationships between tinnitus and mental comorbidities in a methodical manner, with an emphasis on how each disease is assessed and managed in various studies. To be more precise, the objectives are threefold: (1) to identify studies regarding the correlations between tinnitus and mental health disorders; (2) to investigate whether and how tinnitus-specific therapies affect mental symptoms; and (3) to determine whether treating psychiatric comorbidities has quantifiable impacts on tinnitus perception or distress. Our hypothesis is that tinnitus and mental health disorders often co-occur and that treating one may benefit the other.

## 2. Materials and Methods

### 2.1. Search Strategies

A scoping review was carried out to find relevant research on the connection between mental health comorbidities and tinnitus. PubMed, MEDLINE, Embase, PsycINFO, Cochrane Database of Systematic Reviews, and the Google Scholar search engine were the six main electronic databases that were searched between January 2014 and May 2025 to encompass the most recent findings. The review was conducted following the Preferred Reporting Items for Systematic Reviews and Meta-Analyses extension for Scoping Reviews (PRISMA-ScR) standards. To provide comprehensive and inclusive coverage of the tinnitus and mental health domains, search techniques were created using a combination of Subject Headings terms (MeSH, EMTREE, etc.) and free-text keywords. An experienced librarian designed the search strategies (MD). Although the search was designed to be comprehensive across mental health and audiological domains, the focus was on interdisciplinary studies evaluating both psychiatric and tinnitus-related variables. As a result, intervention studies focused solely on tinnitus reduction, such as many CBT and other psychotherapeutic trials that did not include psychiatric-related variables, may not have met the inclusion criteria.

Psychiatric terms, such as depression, anxiety, psychosis, stress, trauma-related symptoms, personality disorders, and sleep difficulties, were included in the search along with the term (and variations) of tinnitus. Phrase variations and thematically similar phrases were captured using proximity operators and Boolean operators. There were no restrictions on the study’s location or environment. The lead author created the search strategy, and a second reviewer checked it for consistency and completeness. Supplementary Material contains comprehensive information about the search approach used for each database. Supplementary Material also contains the PRISMA-ScR checklist that was used to direct the review procedure.

### 2.2. Study Eligibility Criteria

The following criteria were used to determine which studies were included in this review: (1) the main topic was about tinnitus; (2) the study addressed at least one comorbid mental health disorder such as depression, anxiety, stress-related conditions, psychosis, or other psychiatric symptoms; (3) the study described how tinnitus was evaluated or measured (e.g., through self-report, standardized questionnaires, audiological testing, or clinical assessment); (4) the full text was available in English, French or Spanish. Cross-sectional studies, cohort studies, intervention trials, case reports, and theoretical or psychoanalytic writings that offered pertinent therapeutic insights were among the qualitative and quantitative study types that qualified. Furthermore, only studies that were produced between January 2014 to May 2025 were included to reflect the recent state of the literature on this topic. Studies that did not include mental health outcomes, did not describe the procedure for evaluating tinnitus, or did not focus on tinnitus as the primary condition under examination were not considered. The review did not include non-peer-reviewed publications, editorials, dissertations, or gray literature.

### 2.3. Data Extraction

A standardized form created in Microsoft Excel (version 17.0) was used for data extraction, and two authors (VA and JL) independently checked it to guarantee accuracy and consistency. The reviewers discussed and came to a consensus on any differences in the retrieved data or study inclusion. The first author and the year of publication, population/sample characteristics, the method used to assess tinnitus, the mental health comorbidities discussed, the nature or strength of the association between tinnitus and psychiatric symptoms, the type of treatment described for tinnitus, the type of treatment described for psychiatric comorbidities, and the primary outcomes reported were all variables that were methodically extracted from each study. When available, extra notes or remarks pertaining to interpretation or methodological issues were noted. The evidence was consistently documented due to this systematic extraction method, which also facilitated thematic analysis across various study types.

### 2.4. Quality Assessment

A combined approach was employed to appraise the quality of the included studies, drawing from the Mixed Methods Appraisal Tool (MMAT, 2018) and the Joanna Briggs Institute (JBI) Critical Appraisal Tools. The decision to proceed with this mixed approach stems from the methodological diversity of the included studies, which ranged from cross-sectional and cohort designs to clinical case reports, psychological intervention trials, and theoretical essays.

A validated instrument created especially for evaluating complicated and mixed-methods reviews with a variety of qualitative, quantitative, and mixed designs is the MMAT (2018) [18]. Qualitative research randomized controlled trials, non-randomized studies, quantitative descriptive studies, and mixed-methods studies are the five categories of study designs for which it offers standardized checklists. Relevant criteria, such as the suitability of the research questions and study design, the caliber and applicability of data collection techniques, the strength of analytical methods, the use of validated outcome measures, and the consideration of bias and confounding, were evaluated for each study. Studies were grouped according to the number of criteria satisfied, with each criterion being assessed as “Yes”, “No”, or “Can’t tell”.

Relevant checklists from the JBI Critical Appraisal Tools were also used to supplement the MMAT and take into consideration some designs that are not covered by the MMAT (such as case reports and psychoanalytic essays) [19]. With checklists for case reports, text and opinion papers, prevalence studies, and quasi-experimental designs, among other study types, the JBI tools are extensively utilized in evidence synthesis. Items like the case description’s clarity, the clinical judgments’ justification, and the identification of lessons learned were evaluated for case reports. Clear argumentation, practical applicability, and the inclusion of supporting evidence were taken into consideration for theoretical or opinion pieces.

A flexible yet methodical approach to evaluating the methodological strengths and limitations across the entire range of included studies was made possible by the combination of MMAT and JBI tools. To come to an agreement on quality categorization, the research team analyzed each study separately and discussed the findings. Studies were classified as having high, moderate, or low methodological quality depending on how well they performed in relation to the relevant criteria. In accordance with the guidelines of the scoping review process, the findings of this evaluation were utilized to contextualize the strength of the body of evidence rather than as exclusion criteria.

## 3. Results

### 3.1. Description of the Identified Studies

**Figure 1:**
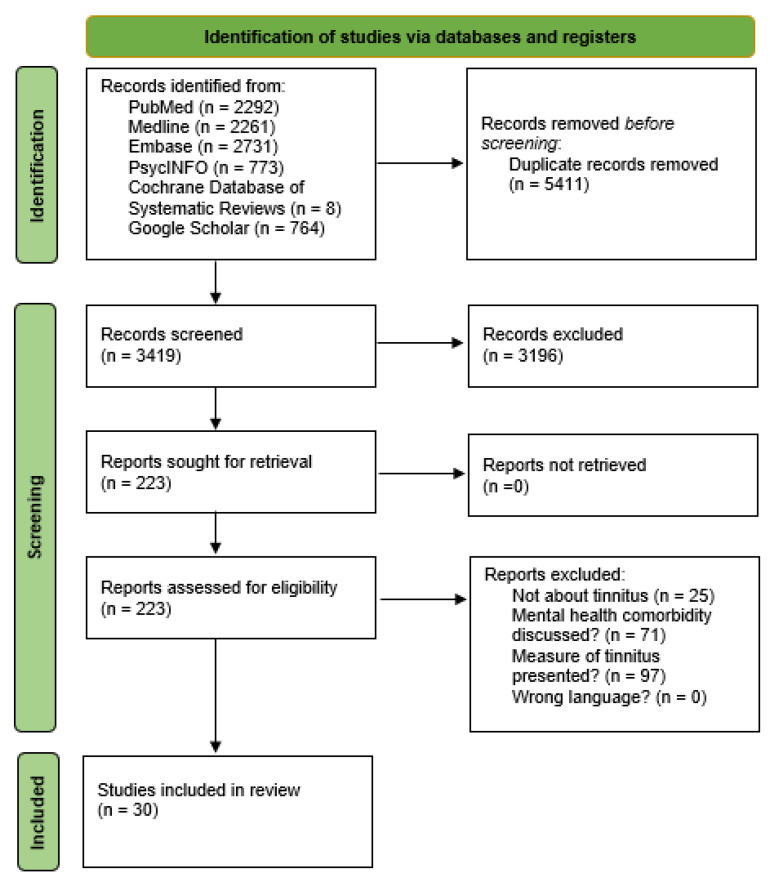
Flowchart of the study selection process.

This scoping review included 30 studies, selected through a comprehensive and systematic search across six databases: PubMed, Medline, Embase, PsycINFO, Cochrane Database of Systematic Reviews, and Google Scholar. A total of 8829 records were initially identified, and after the removal of 5411 duplicates, 3419 records were screened at the title and abstract level. Of these, 3196 were excluded, and 223 full-text articles were assessed for eligibility. Following full-text review, studies were excluded for the following reasons: they were not focused on tinnitus (n = 25), they did not address a psychiatric comorbidity (n = 71), or they did not report how tinnitus was measured (n = 97). No study was excluded due to language restrictions. This process resulted in the final inclusion of 30 eligible studies, as detailed in the PRISMA flow diagram in Figure 1.

**Figure 2:**
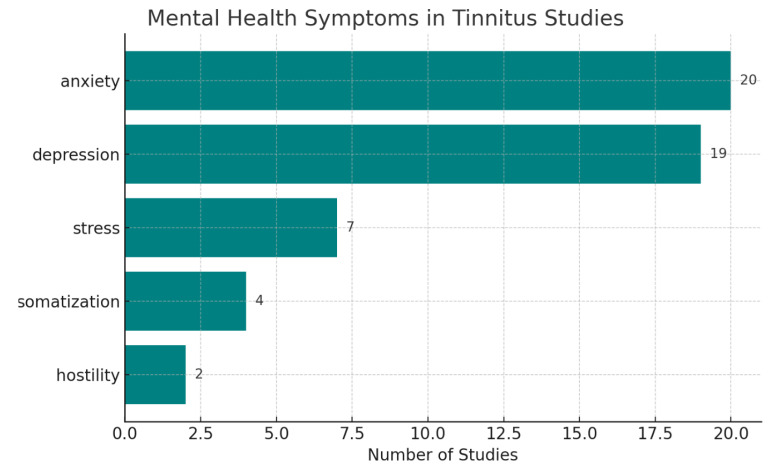
Top five mental health-related symptoms or signs associated with tinnitus in the identified studies.

The included studies represent a diverse range of designs, including cross-sectional and cohort studies, case reports, clinical trials, psychoanalytic essays, and a genome-wide association study (GWAS). Sample sizes varied considerably, ranging from individual case reports to large-scale datasets involving tens of thousands of participants. Tinnitus assessment methods also differed across studies, with the Tinnitus Handicap Inventory (THI) being the most used self-report instrument. Other methods included audiological testing, clinical interviews, and self-reported symptom descriptions. Psychiatric comorbidities were typically evaluated using validated psychological measures, including the Self-Rating Depression Scale (SDS), Self-Rating Anxiety Scale (SAS), and the Hospital Anxiety and Depression Scale (HADS). Top symptoms and signs identified in the studies are found in Figure 2. The full detail of the studies included in the analysis are found in Table S3, also in Supplementary Material.

### 3.2. Associations Between Tinnitus and Psychiatric Comorbidities

All 30 studies included in this review explored, to varying degrees, the relationship between tinnitus and psychiatric symptoms. The most prevalent comorbidities identified were anxiety, depression, stress, and somatization. Numerous cross-sectional studies demonstrated significant associations between tinnitus severity and psychiatric symptomatology. For example, Abbas et al. and Aqeel et al. found strong correlations between tinnitus handicap (THI scores) and depression, anxiety, and stress levels, with gender-specific differences suggesting stronger associations in males [20,21]. Similarly, Han et al. reported that tinnitus severity was significantly correlated with depressive symptoms in both male and female patients, while stress was significantly associated with tinnitus only in males [22].

Population-level evidence supported these findings. Park et al. demonstrated that tinnitus was significantly associated with stress, depressed mood, and suicidal ideation in a nationally representative sample, while Hackenberg et al. found increased odds of depression (OR = 2.03), anxiety (OR = 1.84), and somatization (OR = 2.06) among individuals with tinnitus in the Gutenberg Health Study [23,24]. Other large-scale studies like Bhatt et al. provided genomic evidence of shared genetic pathways between tinnitus and psychiatric conditions, including bipolar disorder and depression [25]. Later work by Park et al. conducted detailed audiological and psychological assessments and confirmed that individuals with normal hearing can nonetheless experience substantial tinnitus-related distress and psychiatric comorbidity [26].

Several studies also emphasized the clinical relevance of psychological subgroups. Beukes et al. conducted a cluster analysis among internet therapy seekers and found that over 60% exhibited moderate to severe depression or anxiety, highlighting the heterogeneity of emotional profiles among tinnitus patients [27]. Chen et al. used network analysis to identify central and bridge symptoms between depression and anxiety, identifying targets like “feeling depressed or hopeless” and “unable to control worry” that could mediate tinnitus distress [28].

Additional studies identified psychiatric symptom patterns within clinical populations. Sahlsten et al. documented both Axis I and Axis II disorders among chronic tinnitus patients, including major depression, OCD, and avoidant personality disorder. Gul et al. [29] and Gomaa et al. confirmed high rates of anxiety, depression, and somatization among patients with chronic tinnitus relative to controls, with stress more closely associated with tinnitus severity than duration [30,31]. Xu et al. also found robust correlations between tinnitus and both depression and anxiety in a large Chinese clinical cohort [32].

Some authors examined the moderating or mediating factors of this relationship. Wang et al. identified that greater resilience, self-esteem, and social support were associated with reduced tinnitus distress [33]. Koning et al. found that peak tinnitus loudness was more strongly correlated with negative affect than mean loudness, emphasizing the role of perceptual variability in psychological outcomes [34].

Psychosis-related symptoms, while less common, were also described in individual case reports. Jain et al. and Alberdi-Páramo et al. presented cases of psychosis and new-onset tinnitus, highlighting the importance of differential diagnosis, particularly when auditory hallucinations are difficult to distinguish from tinnitus. Lucas et al. illustrated how tinnitus could become integrated into a patient’s paranoid delusional system, reinforcing psychiatric decompensation [35,36,37]. Ibraheem et al. added further support to the neuropsychological relevance of tinnitus by demonstrating that individuals with tinnitus, despite normal hearing sensitivity, exhibited impaired temporal resolution and auditory processing, suggesting that subtle central processing abnormalities may underlie distress and perceived severity [38].

Overall, the evidence supports a multifaceted, bidirectional association between tinnitus and a spectrum of psychiatric symptoms, with variability across demographic groups, clinical settings, and tinnitus characteristics.

### 3.3. Tinnitus Treatments and Their Impact on Psychiatric Comorbidities

Several intervention studies evaluated how tinnitus-targeted treatments may improve psychiatric symptoms. CBT emerged as the most commonly studied modality. Weise et al. reported large effects of internet-delivered CBT on tinnitus distress (THI, g = 0.83), with moderate improvements in anxiety, depression, and insomnia [39]. Similar findings were observed by Marks et al., whose randomized controlled trial showed that CBT for insomnia (CBTi) significantly reduced tinnitus distress, insomnia severity, and comorbid depression and anxiety symptoms, with effects sustained at 6-month follow-up [40].

McKenna et al. demonstrated that mindfulness-based cognitive therapy (MBCT) not only reduced tinnitus distress but also yielded significant improvements in anxiety, depression, and avoidance behaviors [41]. Compared to relaxation training, MBCT produced broader and more enduring changes, supporting its use as a psychologically integrative intervention.

Other therapeutic modalities also showed promise. Luyten et al. compared bimodal therapy combining tinnitus retraining therapy (TRT) with either CBT or EMDR [42]. Both approaches significantly reduced tinnitus distress and comorbid anxiety and depression, with EMDR showing continued improvements in some subdomains at follow-up. Moore et al. likewise found that EMDR significantly reduced tinnitus distress and depression in a small clinical sample, though effects on anxiety were not sustained [43].

Crocetti et al. investigated psychophysiological adaptation mechanisms during TRT and found that reductions in tinnitus severity were paralleled by improvements in anxiety, depression, and general psychological well-being [44]. Their findings emphasize the importance of considering emotional reactivity and stress regulation in tinnitus treatment.

Somatic interventions also yielded partial psychiatric benefits. Ketterer et al. reported that cochlear implantation in patients with asymmetric hearing loss reduced tinnitus burden and improved speech perception, although effects on anxiety and depression were minimal [45]. Conversely, studies by Frederiksen et al. and Liu et al. contextualized tinnitus within occupational and environmental stress exposures, noting elevated psychological distress in noisy or extreme work environments, although direct treatment effects were not explored [46,47]. These findings suggest that tinnitus-specific interventions (particularly those grounded in CBT, MBCT, and EMDR) can produce meaningful improvements in psychiatric comorbidities, especially when therapy is individualized and accounts for emotional coping styles.

### 3.4. Psychiatric Comorbidities Treatments and Their Impact on Tinnitus 

Few studies examined whether targeting psychiatric symptoms directly could improve tinnitus. However, clinical reports and conceptual papers attempted to address this gap. Jain et al. presented a case in which trifluoperazine (an antipsychotic) led to improvements in both psychosis and tinnitus symptoms, suggesting a shared dopaminergic mechanism [35]. Alberdi-Páramo et al. also reported a case of new-onset psychosis with tinnitus symptoms that improved following treatment with risperidone [36].

Lucas et al. provided a psychoanalytic case study illustrating how tinnitus became a central element of a patient’s psychotic delusions and unresolved intrapsychic conflict [37]. Despite years of antipsychotic trials, psychoanalytic psychotherapy was credited with helping the patient gain insight and partial symptom relief. Similarly, Papazian interpreted tinnitus as a symbolic response to early life trauma and emotional separation, suggesting a role for psychodynamic conceptualizations in care planning [48]. Salviati et al. suggested a psychosomatic model and found high co-prevalence of anxiety, depression, and personality symptoms in tinnitus patients, advocating for integrated psychiatric support [49].

While these contributions are largely theoretical or based on individual cases, they underline the potential value of psychiatric treatments in alleviating tinnitus distress, particularly when symptoms are embedded in broader psychopathology.

### 3.5. Quality Assessment of the Identified Studies

Of the 30 studies included in this review, 12 were rated as high quality, 13 as moderate, and 5 as low quality. High-quality studies were characterized by robust methodology, validated assessment tools, appropriate statistical analyses, and transparent reporting. These included clinical trials, large-scale cohorts, or genetic and controlled therapeutic evaluations [23,25,39,40,41,42,43]. Wang et al. also stood out for its large sample and strong psychometric modeling of psychosocial factors, while Sahlsten et al. utilized structured psychiatric interviews to assess Axis I and II disorders [29,33].

Moderate-quality studies primarily consisted of cross-sectional or observational designs that, while often employing validated instruments and reporting statistically significant findings, had limitations such as lack of control groups, reduced generalizability, or insufficient longitudinal data [20,31,34,44]. These studies provided valuable correlational insights into the links between tinnitus and mental health comorbidities. Others, such as Frederiksen et al., contributed to contextual understanding but were limited by reliance on self-reported symptoms and absence of objective clinical data [46].

Five studies were rated as low quality due to methodological limitations such as small sample sizes, lack of validated instruments, or anecdotal nature. These included interpretive case reports or psychoanalytic discussions [35,36,37,48]. While not generalizable, these studies offered important clinical insights into psychosis-related tinnitus, symbolic interpretations, or underexplored phenomenological aspects of the condition. Ibraheem et al. also received a low-to-moderate rating due to its very small sample and limited psychiatric focus [38].

## 4. Discussion

### 4.1. Principal Results and Comparison with Prior Works

This scoping review sought to systematically identify the recent literature on the associations between tinnitus and psychiatric comorbidities, with specific attention to how each condition is assessed and whether treatment of one influences the other. Thirty peer-reviewed studies published between 2014 and 2024 were included, encompassing a range of methodologies including observational designs, randomized controlled trials, case reports, and theoretical essays. Across this diverse body of evidence, a consistent finding emerged: tinnitus frequently co-occurs with psychiatric symptoms (most notably anxiety, depression, and stress). Many studies further investigated whether tinnitus-targeted treatments resulted in secondary improvements in psychiatric symptoms, while others explored whether treating psychiatric disorders (e.g., with antipsychotics, SSRIs, or psychotherapy) contributed to reductions in tinnitus severity or distress. Overall, the methodological quality of the studies ranged from moderate to high, with only a minority limited by anecdotal or non-systematic approaches. The findings highlight the bidirectional relationship between tinnitus and mental health and highlight the clinical value of integrated, interdisciplinary management strategies.

The results of this review align with the emerging literature that repositions tinnitus as a condition with significant neuropsychiatric components. Earlier studies often emphasized the audiological dimension of tinnitus, but more recent work highlights the affective, cognitive, and behavioral processes that modulate tinnitus perception and distress. For example, Aazh and Moore reported that more than 80% of patients referred to a tinnitus clinic presented with clinically significant anxiety or depression, while Seydel et al. found that depressive symptoms predicted tinnitus severity more accurately than audiometric variables [43,50,51]. These findings support the routine screening of psychiatric symptoms in tinnitus care settings and corroborate our review’s conclusion that stress, anxiety, and depression are the most prevalent and consequential comorbidities.

There is also evidence supporting the effectiveness of psychological interventions in mitigating tinnitus distress. Among the most well-established approaches, CBT has demonstrated robust outcomes across multiple trials. Weise et al. showed that internet-delivered CBT significantly reduced tinnitus-related distress and emotional symptoms, while Jasper et al. found comparable benefits from both guided self-help and group CBT formats [39,52]. Mindfulness-based approaches, such as MBCT, have also yielded positive effects; McKenna et al. observed significant improvements in both tinnitus severity and comorbid psychological distress following an 8-week MBCT intervention [41]. Sleep disturbance has been effectively addressed through CBTi. Similarly, in a randomized trial, Marks et al. demonstrated that CBTi led to clinically meaningful reductions in insomnia and psychological burden among patients with tinnitus [40]. Beyond traditional CBT, newer multimodal strategies are emerging. Luyten et al. reported that combining CBT or EMDR with TRT significantly improved both tinnitus distress and emotional functioning, with EMDR offering longer-term gains in some domains [42]. EMDR alone also showed promise in reducing tinnitus distress and depressive symptoms, as seen in the study by Moore et al. [43]. These findings align with the current review’s inclusion of studies supporting psychotherapeutic modalities that extend beyond purely audiological interventions.

Although fewer in number, some studies suggest that targeting psychiatric conditions directly may improve tinnitus. Ziai et al. found that SSRIs prescribed for depression were associated with reduced tinnitus intrusiveness [53]. Similarly, Salazar et al. reported decreased tinnitus severity in patients undergoing psychiatric treatment for PTSD, indicating that trauma-focused care may yield ancillary benefits for tinnitus symptoms [54]. While these findings remain preliminary, they echo the present review’s observation that addressing psychiatric comorbidities (particularly in complex or treatment-resistant cases) may be therapeutically meaningful. However, no randomized controlled trials to date have evaluated psychiatric interventions as a primary strategy for tinnitus management, marking an important gap in the literature.

Contemporary conceptual models have also expanded the understanding of tinnitus as a disorder of network dysfunction, implicating auditory, limbic, and prefrontal systems. The recently proposed definition of “Tinnitus Disorder” reflects this evolution, describing not only the auditory percept but also associated emotional, cognitive, and somatic disturbances [55,56]. Stress, in particular, has emerged as both a precipitating and perpetuating factor in tinnitus chronification, reinforcing the value of biopsychosocial assessment frameworks [57]. TRT, which integrates sound therapy and directive counseling, has shown promise in modulating both perceptual and affective components. Meanwhile, neuromodulation techniques (including bimodal stimulation, transcranial magnetic stimulation, and transcranial direct current stimulation) are under investigation for their potential to recalibrate maladaptive neural plasticity. While findings remain variable, these approaches exemplify a shift toward targeting the central nervous system rather than the auditory system alone.

In sum, this review underscores the need to conceptualize and manage tinnitus within a comprehensive, multidisciplinary framework. Affective and cognitive symptoms are not merely secondary consequences of tinnitus, as they are integral to its persistence and clinical burden. Future research should prioritize trials of psychiatric treatments for tinnitus and further investigate shared mechanisms underlying co-occurrence. Until then, integrated care models that simultaneously address tinnitus perception and psychological functioning appear to remain best practice.

### 4.2. Limitations

There are several limitations to consider in this review. While a structured and comprehensive search strategy was implemented across six major databases, the review was intentionally focused on studies that examined both tinnitus and psychiatric comorbidities. As a result, some high-quality intervention studies (particularly randomized controlled trials that assessed tinnitus distress without concurrently measuring psychiatric outcomes) may not have been included. This exclusion may have limited the comprehensiveness of the review with respect to established interventions such as CBT, tinnitus retraining therapy, and mindfulness-based therapies. Additionally, despite efforts to ensure search sensitivity, relevant gray literature or studies published in languages other than English, French, or Spanish may have been overlooked. Methodological heterogeneity across included studies also limited our ability to synthesize findings through meta-analysis or draw strong comparative conclusions. While we conducted a structured quality appraisal using MMAT and JBI tools, the interpretability of certain results remains constrained by the inclusion of lower-quality and highly diverse study designs. Finally, as with all scoping reviews, the descriptive and exploratory nature of the methodology precludes definitive causal inferences regarding the bidirectional relationship between tinnitus and psychiatric comorbidities or the efficacy of specific treatment modalities.

## 5. Conclusions

A thorough summary of the recent studies on the associations between tinnitus and mental comorbidities is given by this scoping review. The results show that people with tinnitus often co-report mental health illnesses like anxiety, sadness, stress, sleeplessness, and, in certain situations, psychosis. The mechanisms behind these correlations, which involve both auditory and non-auditory brain networks, are still complicated and multifactorial, even though these associations are well supported by a variety of study designs. The analysis shows that although tinnitus therapies like EMDR, cochlear implantation, and psychological therapy may have some positive effects on mental health, there is relatively little data on whether psychiatric treatment can reduce tinnitus symptoms specifically. This is indicative of a larger research gap in integrated, bidirectional treatment. The necessity for uniform diagnostic and outcome measurements is highlighted by the fact that different research has different methods for evaluating tinnitus and mental symptoms, which makes comparisons even more difficult. In the future, creating holistic treatment models will require interdisciplinary research that connects clinical psychology, psychiatry, and audiology. While this scoping review applied clear and predefined eligibility criteria to ensure methodological and thematic coherence, we acknowledge that the intentional inclusion of studies focused specifically on tinnitus with clinically measured psychiatric comorbidities may have excluded other relevant literature. This selective scope reflects our aim to synthesize evidence at the intersection of tinnitus and diagnosable mental disorders, and not the full spectrum of tinnitus-related psychological distress. Longitudinal and interventional studies are necessary to better understand how treating one illness may affect the other and to provide evidence-based, patient-centered care methods for people who have both tinnitus and psychological distress.

## Supplementary Materials

The following supporting information can be downloaded at: https://www.mdpi.com/article/10.3390/jcm14113785/s1, Table S1: Electronic search strategy for the scoping review conducted. Table S2: Preferred Reporting Items for Systematic reviews and Meta-Analyses extension for Scoping Reviews (PRISMA-ScR) Checklist. Table S3: Details of the studies included in the analysis.

## Abbreviations

The following abbreviations are used in this manuscript:

CBT	Cognitive behavioral therapy
CBTi	CBT for insomnia
EMDR	Eye movement desensitization and reprocessing
ENT	Ear, Nose, Throat
GWAS	Genome-wide association study
HADS	Hospital Anxiety and Depression Scale
JBI	Joanna Briggs Institute
MMAT	Mixed Methods Appraisal Tool
PRISMA-ScR	Preferred Reporting Items for Systematic Reviews and Meta-Analyses extension for Scoping Reviews
PTSD	Post-traumatic stress disorder
SAS	Self-Rating Anxiety Scale
SDS	Self-Rating Depression Scale
SSRI	Selective Serotonin Reuptake Inhibitor
THI	Tinnitus Handicap Inventory
