# Supplementary material for: When the Mind Meets the Ear: A Scoping Review on Tinnitus and Clinically Measured Psychiatric Comorbidities
Source: J Clin Med. 2025 May 28;14(11):3785. doi: 10.3390/jcm14113785 (PMC12155966; doi:10.3390/jcm14113785)
Supplement: Supplementary file 1 [file jcm-14-03785-s001.zip › jcm-3598700-supplementary.pdf]

Supplementary Online Content

Table S1. Electronic search strategy for the scoping review conducted.

Database; Search	Search Terms
PubMed; k= 2292	("Tinnitus"[Mesh] OR tinnitus[TIAB]) AND ("Mental Disorders"[Mesh:NoExp] OR "Mental Health"[Mesh] OR "Affective Symptoms"[Mesh] OR "Stress, Psychological"[Mesh:NoExp] OR "Psychological Distress"[Mesh] OR "Anxiety Disorders"[Mesh] OR "Mood Disorders"[Mesh] OR "Personality Disorders"[Mesh] OR "Schizophrenia Spectrum and Other Psychotic Disorders"[Mesh] OR "Somatoform Disorders"[Mesh] OR "Trauma and Stressor Related Disorders"[Mesh] OR "Sleep Wake Disorders"[Mesh] OR Psychiatric[TIAB] OR "Mental disorder*"[TIAB] OR "Mental illness*"[TIAB] OR "Mental health"[TIAB] OR "Psychological disorder"[TIAB:~3] OR "psychological disorders"[TIAB:~3] OR "Psychological problem"[TIAB:~3] OR "psychological problems"[TIAB:~3] OR "Psychological symptom"[TIAB:~3] OR "psychological symptoms"[TIAB:~3] OR "Psychological disturbance"[TIAB:~3] OR "psychological disturbances"[TIAB:~3] OR "emotional disorder"[TIAB:~3] OR "emotional disorders"[TIAB:~3] OR "emotional problem"[TIAB:~3] OR "emotional problems"[TIAB:~3] OR "emotional symptom"[TIAB:~3] OR "emotional symptoms"[TIAB:~3] OR "emotional disturbance"[TIAB:~3] OR "emotional disturbances"[TIAB:~3] OR "psychological distress"[TIAB:~3] OR "emotional distress"[TIAB:~3] OR Anxiety[TIAB] OR Phobia*[TIAB] OR Phobic[TIAB] OR "Obsessive compulsive"[TIAB] OR "Personality disorder*"[TIAB] OR Depression[TIAB] OR Depressive[TIAB] OR "Affective disorder*"[TIAB] OR "affective symptom*"[TIAB] OR "Mood disorder*"[TIAB] OR "mood symptom*"[TIAB] OR "Bipolar disorder"[TIAB:~2] OR "bipolar disorders"[TIAB:~2] OR Psychosis[TIAB] OR Psychotic[TIAB] OR Schizo*[TIAB] OR Somatization[TIAB] OR somatisation[TIAB] OR "Somatic symptom*"[TIAB] OR "Somatic disorder*"[TIAB] OR Somatoform[TIAB] OR "Body concept"[TIAB] OR "Body dysmorphic"[TIAB] OR "psychological stress"[TIAB:~3] OR "emotional stress"[TIAB:~3] OR "stress disorder*"[TIAB] OR "mental stress"[TIAB] OR "stress problem"[TIAB:~3] OR "stress problems"[TIAB:~3] OR "problematic stress"[TIAB:~3] OR "stress-related"[TIAB] OR "Post-traumatic"[TIAB] OR Posttraumatic[TIAB] OR PTSD[TIAB] OR "sleep disorder"[TIAB:~3] OR "sleep disorders"[TIAB:~3] OR "sleeping disorder"[TIAB:~3] OR "sleeping disorders"[TIAB:~3] OR "sleep problem"[TIAB:~3] OR "sleep problems"[TIAB:~3] OR "problematic sleep"[TIAB:~3] OR "sleeping problem"[TIAB:~3] OR "sleeping problems"[TIAB:~3] OR "problematic sleeping"[TIAB:~3] OR Insomnia*[TIAB])
Medline; k= 2261	1. Tinnitus/<br>2. tinnitus.ti,ab,kw.<br>3. or/1-2<br>4. Mental Disorders/<br>5. Mental Health/<br>6. Affective Symptoms/<br>7. Stress, Psychological/<br>8. exp Psychological Distress/<br>9. exp anxiety disorders/ or exp obsessive-compulsive disorder/ or exp phobic disorders/

	10. exp mood disorders/ or exp "bipolar and related disorders"/ or exp depressive disorder/<br>11. exp Personality Disorders/<br>12. exp "schizophrenia spectrum and other psychotic disorders"/ or exp psychotic disorders/ or exp schizophrenia/<br>13. exp Somatoform Disorders/<br>14. exp "trauma and stressor related disorders"/ or exp stress disorders, traumatic/<br>15. exp sleep wake disorders/ or exp dyssomnias/ or exp sleep disorders, intrinsic/ or exp parasomnias/<br>16. (Psychiatric or "Mental disorder*" or "Mental illness*" or "Mental health" or ((psychological or emotional) adj3 (disorder* or problem* or symptom* or disturbance* or distress)) or Anxiety or Phobia* or Phobic or "Obsessive compulsive" or "Personality disorder*" or Depression or Depressive or "Affective disorder*" or "affective symptom*" or "Mood disorder*" or "mood symptom*" or (bipolar adj2 disorder*) or Psychosis or Psychotic or Schizo* or Somati#ation or "Somatic symptom*" or "Somatic disorder*" or Somatoform or "Body concept" or "Body dysmorphic" or ((psychological or emotional) adj3 stress) or "stress disorder*" or "mental stress" or (stress adj3 problem*) or "stress-related" or "Post-traumatic" or Posttraumatic or PTSD or (sleep* adj3 (disorder* or problem*)) or Insomnia*).ti,ab,kw.<br>17. or/4-16<br>18. and/3,17
Embase; k = 2731	1. *tinnitus/<br>2. tinnitus.ti,ab,kw.<br>3. or/1-2<br>4. mental disease/<br>5. mental health/<br>6. exp emotional disorder/<br>7. mental stress/ or emotional stress/<br>8. distress syndrome/<br>9. exp anxiety disorder/ or exp obsessive compulsive disorder/ or exp phobia/ or exp posttraumatic stress disorder/<br>10. exp mood disorder/ or exp depression/ or exp mania/<br>11. exp personality disorder/ or exp cluster a personality disorder/ or exp cluster b personality disorder/ or exp cluster c personality disorder/<br>12. exp schizophrenia spectrum disorder/ or exp schizophrenia/<br>13. exp psychosis/<br>14. exp psychosomatic disorder/ or exp somatoform disorder/<br>15. exp body dysmorphic disorder/<br>16. sleep disorder/ or exp abnormal dreaming/ or exp dyssomnia/ or exp parasomnia/ or exp sleep misperception/ or exp somnolence/<br>17. (Psychiatric or "Mental disorder*" or "Mental illness*" or "Mental health" or ((psychological or emotional) adj3 (disorder* or problem* or symptom* or disturbance* or distress)) or Anxiety or Phobia* or Phobic or "Obsessive compulsive" or "Personality disorder*" or Depression or Depressive or "Affective disorder*" or "affective symptom*" or "Mood disorder*" or "mood symptom*" or (bipolar adj2 disorder*) or Psychosis or Psychotic or Schizo* or Somati#ation or "Somatic symptom*" or "Somatic disorder*" or Somatoform or "Body concept" or "Body

	dysmorphic" or ((psychological or emotional) adj3 stress) or "stress disorder*" or "mental stress" or (stress adj3 problem*) or "stress-related" or "Post-traumatic" or Posttraumatic or PTSD or (sleep* adj3 (disorder* or problem*)) or Insomnia*).ti,ab,kw.<br>18. or/4-17<br>19. and/3,18<br>20. limit 19 to (article or article in press or "review")
PsycINFO; k= 773	1. tinnitus/<br>2. tinnitus.ti,ab,id.<br>3. or/1-2<br>4. mental disorders/<br>5. mental health/<br>6. exp psychiatric symptoms/ or exp anhedonia/ or exp anxiety/ or exp hallucinations/ or exp mania/<br>7. psychological stress/<br>8. distress/<br>9. exp anxiety disorders/ or exp phobias/ or exp obsessive compulsive disorder/<br>10. exp affective disorders/ or exp major depression/ or exp persistent depressive disorder/ or emotional disturbances/<br>11. exp personality disorders/<br>12. exp psychosis/ or exp schizophrenia/<br>13. exp somatoform disorders/ or exp body dysmorphic disorder/<br>14. exp "stress and trauma related disorders"/ or exp posttraumatic stress disorder/ or posttraumatic stress/<br>15. exp sleep wake disorders/ or exp parasomnias/<br>16. (Psychiatric or "Mental disorder*" or "Mental illness*" or "Mental health" or ((psychological or emotional) adj3 (disorder* or problem* or symptom* or disturbance* or distress)) or Anxiety or Phobia* or Phobic or "Obsessive compulsive" or "Personality disorder*" or Depression or Depressive or "Affective disorder*" or "affective symptom*" or "Mood disorder*" or "mood symptom*" or (bipolar adj2 disorder*) or Psychosis or Psychotic or Schizo* or Somati#ation or "Somatic symptom*" or "Somatic disorder*" or Somatoform or "Body concept" or "Body dysmorphic" or ((psychological or emotional) adj3 stress) or "stress disorder*" or "mental stress" or (stress adj3 problem*) or "stress-related" or "Post-traumatic" or Posttraumatic or PTSD or (sleep* adj3 (disorder* or problem*)) or Insomnia*).ti,ab,id.<br>17. or/4-16<br>18. and/3,17<br>19. limit 18 to "0100 journal"
Cochrane Database of Systematic Reviews; k= 8	1. tinnitus.ti,ab.<br>2. (Psychiatric or "Mental disorder*" or "Mental illness*" or "Mental health" or ((psychological or emotional) adj3 (disorder* or problem* or symptom* or disturbance* or distress)) or Anxiety or Phobia* or Phobic or "Obsessive compulsive" or "Personality disorder*" or Depression or Depressive or "Affective disorder*" or "affective symptom*" or "Mood disorder*" or "mood symptom*" or (bipolar adj2 disorder*) or Psychosis or Psychotic or Schizo* or Somati#ation or "Somatic symptom*" or "Somatic disorder*" or Somatoform or "Body concept" or "Body dysmorphic" or ((psychological or emotional) adj3 stress) or "stress disorder*" or

	"mental stress" or (stress adj3 problem*) or "stress-related" or "Post-traumatic" or Posttraumatic or PTSD or (sleep* adj3 (disorder* or problem*)) or Insomnia*).ti,ab.<br>3. and/1-2
Google Scholar; k= 764	"tinnitus" AND ("psychiatric comorbidity" OR "psychological distress") AND ("tinnitus treatment" OR "mental health intervention") after:2014

Table S2. Preferred Reporting Items for Systematic reviews and Meta-Analyses extension for Scoping Reviews (PRISMA-ScR) Checklist

SECTION	ITEM	PRISMA-ScR CHECKLIST ITEM	REPORTED ON PAGE #
**TITLE**			
Title	1	Identify the report as a scoping review.	1
**ABSTRACT**			
Structured summary	2	Provide a structured summary that includes (as applicable): background, objectives, eligibility criteria, sources of evidence, charting methods, results, and conclusions that relate to the review questions and objectives.	1
**INTRODUCTION**			
Rationale	3	Describe the rationale for the review in the context of what is already known. Explain why the review questions/objectives lend themselves to a scoping review approach.	2
Objectives	4	Provide an explicit statement of the questions and objectives being addressed with reference to their key elements (e.g., population or participants, concepts, and context) or other relevant key elements used to conceptualize the review questions and/or objectives.	2,3
**METHODS**			
Protocol and registration	5	Indicate whether a review protocol exists; state if and where it can be accessed (e.g., a Web address); and if available, provide registration information, including the registration number.	N/A
Eligibility criteria	6	Specify characteristics of the sources of evidence used as eligibility criteria (e.g., years considered, language, and publication status), and provide a rationale.	3
Information sources*	7	Describe all information sources in the search (e.g., databases with dates of coverage and contact with authors to identify additional sources), as well as the date the most recent search was executed.	3
Search	8	Present the full electronic search strategy for at least 1 database, including any limits used, such that it could be repeated.	3
Selection of sources of evidence†	9	State the process for selecting sources of evidence (i.e., screening and eligibility) included in the scoping review.	3
Data charting process‡	10	Describe the methods of charting data from the included sources of evidence (e.g., calibrated forms or forms that have been tested by the team before their use, and whether data charting was done independently or in duplicate) and any processes for obtaining and confirming data from investigators.	4
Data items	11	List and define all variables for which data were sought and any assumptions and simplifications made.	4
Critical appraisal of individual sources of evidence§	12	If done, provide a rationale for conducting a critical appraisal of included sources of evidence; describe the methods used and how this information was used in any data synthesis (if appropriate).	4

SECTION	ITEM	PRISMA-ScR CHECKLIST ITEM	REPORTED ON PAGE #
Synthesis of results	13	Describe the methods of handling and summarizing the data that were charted.	5
**RESULTS**			
Selection of sources of evidence	14	Give numbers of sources of evidence screened, assessed for eligibility, and included in the review, with reasons for exclusions at each stage, ideally using a flow diagram.	5,6
Characteristics of sources of evidence	15	For each source of evidence, present characteristics for which data were charted and provide the citations.	7,28
Critical appraisal within sources of evidence	16	If done, present data on critical appraisal of included sources of evidence (see item 12).	7,28
Results of individual sources of evidence	17	For each included source of evidence, present the relevant data that were charted that relate to the review questions and objectives.	7,28
Synthesis of results	18	Summarize and/or present the charting results as they relate to the review questions and objectives.	7,28
**DISCUSSION**			
Summary of evidence	19	Summarize the main results (including an overview of concepts, themes, and types of evidence available), link to the review questions and objectives, and consider the relevance to key groups.	29
Limitations	20	Discuss the limitations of the scoping review process.	30
Conclusions	21	Provide a general interpretation of the results with respect to the review questions and objectives, as well as potential implications and/or next steps.	30
**FUNDING**			
Funding	22	Describe sources of funding for the included sources of evidence, as well as sources of funding for the scoping review. Describe the role of the funders of the scoping review.	31

JBI = Joanna Briggs Institute; PRISMA-ScR = Preferred Reporting Items for Systematic reviews and Meta-Analyses extension for Scoping Reviews.
* Where *sources of evidence* (see second footnote) are compiled from, such as bibliographic databases, social media platforms, and Web sites.
† A more inclusive/heterogeneous term used to account for the different types of evidence or data sources (e.g., quantitative and/or qualitative research, expert opinion, and policy documents) that may be eligible in a scoping review as opposed to only studies. This is not to be confused with *information sources* (see first footnote).
‡ The frameworks by Arksey and O'Malley (6) and Levac and colleagues (7) and the JBI guidance (4, 5) refer to the process of data extraction in a scoping review as data charting.
§ The process of systematically examining research evidence to assess its validity, results, and relevance before using it to inform a decision. This term is used for items 12 and 19 instead of "risk of bias" (which is more applicable to systematic reviews of interventions) to include and acknowledge the various sources of evidence that may be used in a scoping review (e.g., quantitative and/or qualitative research, expert opinion, and policy document).

From: Tricco AC, Lillie E, Zarin W, O'Brien KK, Colquhoun H, Levac D, et al. PRISMA Extension for Scoping Reviews (PRISMAScR): Checklist and Explanation. Ann Intern Med. 2018;169:467–473. doi: 10.7326/M18-0850.

Table S3. Details of the studies included in the analysis.

Authors	Population	Tinnitus Evaluation	Psychiatric Comorbidities	Strength of Association	Tinnitus Treatment	Psychiatric Treatment	Main Outcomes	Quality assessment
Abbas et al., 2019 [20]	100 adult patients (60 males 40 females) with chronic tinnitus from various ENT and audiology departments in public and private hospitals in Pakistan.	Tinnitus handicap inventory (THI) (25 items scale), audiometer, otoscope (to rule out biological symptoms), air and bone conduction and tympanometry examination.	Anxiety, stress, depression, mood swings.	Spearman's test, tinnitus handicap index (THI) is associated with 0. 59 correlation with dass (depression anxiety stress scale)(p<0. 001), 0. 62 correlation with anxiety (p<0.001), 0. 55 correlation with depression (p<0.01), in male patients, tinnitus was more strongly associated to anxiety (0. 66, p<0.001) and depression (0. 59, p<0.001), whereas female patients did not show such association. on the	N/A	N/A	The authors reported a significant association between tinnitus and psychological problems, especially in male patients. in female patients, tinnitus was found to mediate positive affect/negative affect.	Moderate

				other hand, female patients reported a significant associatio n between tinnitus and positive affects (0. 80, p<0.001).				

Alberdi-Paramo et la., 2018 [36]	Case report of a 17 year-old female patient presenting at the emergency for worsening bilateral tinnitus for 3 months.	Self-reported. the patient visited multiple times to the ed for worsening tinnitus bilaterally. audiology, ear, nose, and throat and neurology tests were done, revealing no abnormality. frequency and intensity of tinnitus was modified according to certain circumstances like surrounding noise or posture. associated symptoms were contracture and pain in the jaw and face and changes in the tone of the voice., patient also presented with behavioral alteration and suicidal thoughts, which	(early onset) psychosis	N/A	Tylenol and dilaudid were given to the patient at the ED before the diagnosis was given.	Risperdal (2 mg, oral administration).	Tinnitus may be a symptom associated with a psychotic disorder. careful tests, questionnaires and examinations of a patient presenting with tinnitus is warranted in order to reveal hints of atypical tinnitus features and psychosis symptoms., in certain cases where tinnitus may be the only symptom and the diagnostic of a psychosis is not clear, the authors propose to initiate an antipsychotic treatment.	Moderate

		ultimately led to a psychiatry evaluation and diagnosis of a psychosis.							

Aqeel et al., 2017 [21]	110 patients (70 males, 40 females) were sampled from ENT and audiology department from clinics and hospitals in urban cities in Pakistan. The sampling was based on a correlation sampling design (Purposive sampling technique).	Audiology test, THI.	Depression, anxiety, stress.	THI had significant correlations with: depression (0.555, p<0.01), anxiety (0.634, p<0.01), stress (0.548, p<0.01), hearing loss (0.812, p<0.001), hearing loss was positively correlated with, depression (0.492, p<0. 001), stress (0.497, p<0. 001), anxiety (0.531, p<0.001), tinnitus perception (THI), hearing loss and psychological problems (depression, stress and anxiety) were all statistically significative more prevalent in female tinnitus than in male	N/A	N/A	Hearing loss is positively correlated with tinnitus perception and psychological problems in people with tinnitus complaints.	High

				tinnitus. In male tinnitus patients, tinnitus was significantly associated with hearing loss (0.36, p< 0.01), anxiety (0.66, p<0.001), depression (0.59, p<0.001) and stress (0.54, p<0.001). no significant association was found for female tinnitus patients and psychological problems.					

Beukes et al., 2022 [27]	399 adults with bothersome tinnitus seeking internet-based cognitive behavioral therapy.	Self-reported, the severity was assessed using tinnitus functional index.	Anxiety, depression, insomnia.	44.6% had mild to moderate anxiety and 11.03% had severe anxiety. 43.9% had mild to moderate depression and 15.3% had moderate to severe depression, 31.1% had subthreshold insomnia and 36.8% had moderate to severe clinical insomnia, the cluster analysis formed 4 groups:, cluster 1 (24.4%): debilitating tinnitus, associated with severe anxiety ($p=0.0006$), associated with being Hispanic or Latino, cluster 2 (10. 3%): distressing tinnitus, associated with being Hispanic	N/A	N/A	The authors emphasize the importance of attentive analysis of the vocabulary used by patients to describe their tinnitus symptoms is a great tool to characterize the patients into 4 clusters. These clusters and their associations can be used as guidance on the treatment and approach of tinnitus patients.	Low

				or Latino, cluster 3 (46%): annoying tinnitus, associated with no anxiety (p=0.002), - cluster 4 (19.40%): accepting tinnitus.					

Bhatt et al. 2022 [25]	Using the UK Biobank sample comprised of 132 438 individuals with tinnitus and genotype data.	The UK Biobank collected data from volunteers from 2006-2010. Individuals filled a touchscreen questionnaire and one question was "do you hear or have you heard noises (such as ringing or buzzing) in your head or in one or both ears that last for more than five minutes at a time?".	No specific psychiatric comorbidity was mentioned, however the enrichment analysis found genes associated with psychiatric conditions (not specified).	A SNP (GPM6a, ADAM29) achieved significant association with tinnitus genome wide(p<0.001), (GPM6a is associated with psychiatric traits, such as bipolar diseases, schizophrenia, depression, Alzheimer's disease, and claustrophobia), 19 other SNPs suggested association with tinnitus, however did not reach genome-wide significance. The gene-based test showed a significant association between psap and tinnitus-related distress, based on genomic	N/A	N/A	This study identified the main SNPs associated with tinnitus, including GPM6a which revealed to be significant genome-wide, and 19 other SNPs that were suggestive of an association with tinnitus., genomic associations revealed association between tinnitus-related genes and psychiatric conditions.	High

				associations, analysis identified psychiatric, cardiovascular, metabolic, hematological, and pharmacogenomic association with tinnitus. The genome wide association study revealed that the genes associated with tinnitus were upregulated in the hippocampus and cortex.				

Chen et al., 2023 [28]	566 tinnitus sufferers (age range: 11–86 years; ~50% male/female) from the tinnitus outpatient department of Chinese PLA General Hospital, Beijing.	Self-reported tinnitus status; no standardized tinnitus severity index was used beyond general classification (VAS for tinnitus severity). Tinnitus-related variables: onset (gradual/abrupt), duration, location (right, left, bilateral, head), and type (intermittent/constant).	Depression and anxiety assessed using standardized DSM-V-aligned tools: PHQ-9 (depression) and GAD-7 (anxiety). High internal consistency reported (Cronbach's α = 0.90 for PHQ-9, 0.91 for GAD-7). Also investigated suicidal ideation (PHQ-9 item 9).	Network analysis showed strong partial correlations within depression and within anxiety items. Central symptoms with the highest expected influence: "Unable to control worry" (GAD-2), "Relaxation difficulty" (GAD-4), and "Feeling depressed or hopeless" (PHQ-2). Bridge symptoms connecting anxiety and depression: "Afraid something awful might happen" (GAD-7), "Feeling of worthlessness" (PHQ-6), and "Trouble concentrat	N/A – No tinnitus intervention tested in this study; observational cross-sectional study.	N/A – No treatment delivered; however, the authors discuss potential implications for transdiagnostic treatment targets (e.g., CBT, MCT, mindfulness) for symptoms with high centrality.	Key central and bridge symptoms of depression and anxiety were identified in tinnitus sufferers. These symptoms may serve as effective therapeutic targets. "Afraid something awful might happen" and "Feeling depressed or hopeless" were most strongly connected to suicidal ideation, suggesting the need for focused interventions.	Moderate

				ing" (PHQ-7).				
Crocetti et al., 2018 [44]	92 Caucasian patients attending tinnitus retraining therapy in Milan.	ENT evaluation and audiology evaluation to determine etiology of tinnitus using the THI scale.	Stress, anxiety, depression, general discomfort, subjective well being, perception of change.	At T0: tinnitus was significantly correlated (Pearson's) with: anxiety (0.351), subjective well being (-0.403), depression (0.455), general psychological disease (0.424), psychopathological diagnosis	Tinnitus retraining therapy, based on cognitive behavioral concepts., showed significant improvement after 3 months on tinnitus disease and on associated psychological problems.	N/A	Reconditioning methods for tinnitus treatment significantly help with the severity of tinnitus perception. it also supports its benefits on associated psychopathological cal disease.	Moderate

				(0.297), tinnitus severity (THI) decreases significantly in time with the tinnitus retraining therapy at 3months (p<0.01), psychopathological disease significantly influence tinnitus severity (p=0.002), no association was found between tinnitus and psychophysiological arousal.					

Frederiksen et al., 2016 [46]	534 Danish workers with data collected from a survey from 2009-2010.	Tinnitus was described in the survey questionnaire as "ringing or buzzing in one or both ears".	Symptoms of depression, anxiety and somatoform disorder were assessed in the survey using the CMDQ (common mental disorders questionnaire).	For each 10 dB increase, an OR of 0.95 for tinnitus was noted (not significative), no statistically significant association was found between work psychological demands and tinnitus (OR 1. 07, CI:0. 90,1. 26).	N/A	N/A	Occupational noise does not seem to be associated with tinnitus. likewise, psychosocial working conditions are not associated with tinnitus.	Moderate-High
Goomaa et al., 2014 [31]	196 participants: 100 patients with subjective tinnitus and hearing loss, 45 with hearing loss only, and 50 healthy controls. Age range: 20–60 years.	Visual Analogue Scale (VAS) for tinnitus intensity (classified as mild, moderate, or severe); full ENT examination; audiological evaluation including pure tone audiometry, tympanometry, speech discrimination, acoustic reflexes, and ABR.	Depression, anxiety, and stress evaluated via DASS (Depression, Anxiety, Stress Scales – 14 items per subscale). High prevalence in tinnitus group: depression (84%), anxiety (86%), stress (73%). Severity varied by age and gender. Hearing	Strong correlation between **duration of tinnitus** and severity of **depression** (p = 0.02) and **anxiety** (p = 0.05); no correlation with tinnitus severity for depression or anxiety. However, **severity of tinnitus** was	N/A	Authors conclude mental health symptoms must be taken into account in tinnitus management.	Tinnitus patients exhibit markedly elevated rates of depression, anxiety, and stress compared to controls and hearing loss-only participants. Correlations suggest that longer duration and greater tinnitus severity are linked to greater emotional distress, particularly stress. Supports integration of psychological assessment in tinnitus care.	Moderate

			loss group had very low rates of psychiatric symptoms.	significantly correlated with **stress** (p = 0.04). **Age** was significantly associated with all three domains.				
Gul et al., 2015 [30]	50 patients with chronic tinnitus for at least 6 months and 50 healthy volunteers.	Diagnosis was self-reported (patients with a complaint of tinnitus).	Anxiety, somatization, depression.	Scores between the chronic tinnitus group and the control group where significantly different for: anxiety sensitivity index-3 (-8.11, p<0.001), scl-90 (-8.00, p<0.001), scl-90 somatization (-8.22, p<0.001), scl-90	N/A	N/A	Anxiety sensitivity, anxiety and depression were significantly higher in patients with tinnitus than in the control group.	Moderate

				depression (-8.19, p<0.001), scl-90 anxiety (-8.00, p<0.001).					
Hackenberg et al., 2023 [24]	8539 adults from the Gutenberg Health Study (Mainz, Germany), representative of the general population. Mean age: 60.7 years, 51.1% male. Tinnitus point prevalence: 28.0% (n = 2387).	Self-report questions on tinnitus presence and burden (scale 1–6); audiological testing via pure-tone audiometry for hearing loss classification.	Depression (PHQ-9), Anxiety (GAD-7), and Somatic Symptom Disorders (SSS-8). Prevalence among tinnitus group: depression 7.9%, anxiety 5.4%, somatization 40.4%. Higher prevalence than non-tinnitus group across all domains.	Logistic regression: tinnitus associated with higher odds of depression (OR = 2.03), anxiety (OR = 1.84), and somatic symptom disorder (OR = 2.06), all p < 0.0001. Tinnitus burden positively correlated with PHQ-9 (r = 0.15), GAD-7 (r = 0.13), and SSS-8 (r = 0.20).	N/A – Observational cohort; no treatment intervention.	N/A – Psychiatric symptoms measured only; no treatment provided. Authors suggest integrating psychosomatic/ psychotherapy support into tinnitus care.	Tinnitus strongly associated with depressive, anxiety, and somatic symptoms. Somatic symptoms had the strongest independent association. Hearing loss also independently associated with tinnitus. Suggests integrated assessment of psychological distress and hearing in tinnitus patients. Females had higher burden of psychiatric symptoms than males.	High

Han et al., 2019 [22]	134 female and 114 male patients consulting at Seoul St. Mary's Hospital for ENT clinic for tinnitus.	THI, various scales assessing tinnitus characteristics (loudness, annoyance, etc.).	Depression, anxiety.	Tinnitus severity was correlated to depressive symptoms (0.411, p<0.01 in males)(0.304, p<0.01 in females), the correlation between stress and tinnitus was only observed in male patients (0.463, p<0.001), depressive symptoms was associated with tinnitus severity in both men (0.411, p<0.01) and women (0.304, p<0.01).	N/A	N/A	Tinnitus is significantly correlated with depressive symptoms and stress, although there are gender differences.	Moderate
Ibraheem et al., 2016 [38]	15 adults with tinnitus attending audiology unit or ENT department, 15 adults	Level of annoyance and sleep disturbance was noted on a scale from 1-5, tinnitus psychoacoustic measures, transient-evoked otoacoustic	No psychiatric comorbidities were mentioned.	Significantly higher hearing threshold were observed in tinnitus ears (p=0.003), significant elevation of approximate GIN	N/A	N/A	There is an auditory impairment in tinnitus patients. it is recommended to include a temporal resolution testing such as gin in evaluation of tinnitus patients.	Low-Moderate

	without tinnitus.	emissions (TEOASE), and GIN (gaps-in-noise) test.		thresholds (p=0.000) and a reduction of GIN correct scores (p=0.000).				
Jain et al., 2017 [35]	Case report of a male patient diagnosed with schizophrenia at age 38. He presents with tinnitus symptoms at age 48 that are difficult to differentiate with auditory hallucinations. This patient also had a history of bilateral otitis media at age 18.	Self-reported, patient complained of wind blowing sounds. sounds were misinterpreted during psychosis and delusions.	Psychosis.	N/A	Tinnitus responded well to the trifluoperazine and sounds were significantly reduced.	Trifluoperazine, risperidone, olanzapine.	This case study highlights the possible association between middle ear disease, development of psychosis and tinnitus.	Low

Ketterer et al., 2018 [45]	44 patients (24 males, 20 females) suffering from unilateral asymmetric hearing loss.	Tinnitus questionnaire (TQ).	Anxiety, stress, depression, emotional distress and cognitive distress (before and after treatment).	N/A	Cochlear implant. a statistically significant reduction in tinnitus-related distress was observed after cochlear implantation ($p<0.001$).	The study didn't include specific treatments targeting psychiatric comorbidities.	The authors reported that there were no notable improvement in anxiety or depression after installing a cochlear implant.	High
Koning et al., 2019 [34]	82 patients (47 males, 35 females) treated for tinnitus between January 2017 and September 2019.	Evaluated using the visual analogue scale (0-100 mm).	Depression, disturbed concentration, fear and anger (observed after the onset of tinnitus).	Maximal tinnitus loudness : disturbed concentration ($p=0.003$), depression ($p=0.006$), fear ($p=0.014$), anger ($p=0.042$), mean tinnitus loudness : disturbed concentration ($p=0.224$), depression ($p=0.39$), fear ($p=0.964$), anger ($p=0.322$).	The study suggests therapy aimed at reducing the maximal tinnitus loudness to below 73 mm on the visual analogue scale. (treatment not specified).	No direct treatment for psychiatric comorbidities was described. Reduction in tinnitus loudness was proposed to reduce associated psychiatric comorbidities.	Psychiatric comorbidities were strongly associated with the maximal loudness of tinnitus, with no significant association with mean loudness.	Moderate

Liu et al., 2019 [47]	179 workers (179 males, 0 females) from the Erdaogou Mine in China.	Self-reported.	Somatization, compulsion, interpersonal sensitivity, depression, anxiety, hostility, terror, paranoia, psychosis (after working in the mine).	N/A	N/A	The study suggested to improve working conditions.	The study couldn't identify direct correlation between tinnitus and psychiatric comorbidities.	Moderate
Lucas et al., 2020 [37]	Single case study of an adult male.	Self-reported.	Paranoia psychosis, psychotic depression (before and after tinnitus. exacerbated with tinnitus).	N/A	N/A	Antidepressants, antipsychotics, and five years of psychoanalytic psychotherapy (results: the medication helped resolve the patient's depressive state quickly, but the paranoid delusional belief persisted despite trials of several antipsychotics. as for the psychotherapy, it helped increase awareness of internal conflicts, but the delusional belief was not fully resolved).	The authors reported that tinnitus can exacerbate underlying psychiatric vulnerabilities, leading to psychotic depression and/or paranoid delusions.	Low

Luyten et al., 2020 [42]	89 adults with chronic, subjective, non-pulsatile tinnitus (>3 months), aged 18–75.	Tinnitus Functional Index (TFI), Tinnitus Questionnaire (TQ), Visual Analog Scale for tinnitus loudness (VASLoudness), psychoacoustic measures (pitch matching, loudness matching, residual inhibition).	Anxiety and depression assessed using HADS. Hyperacusis assessed using HQ. Exclusion of patients with acute psychiatric comorbidity or HADS >15. Comorbid symptoms common (e.g., depression, anxiety, hypersensitivity to sound).	Both groups showed significant TFI reduction: TRT/CBT (−15.1), TRT/EMDR (−16.2); $p < 0.001$ for both. Anxiety (HADS-A), depression (HADS-D), HQ, and VASLoudness all improved significantly ($p < 0.001$). No significant difference between groups overall, but TRT/EMDR showed continued improvement at follow-up in some subscales (e.g., relaxation).	Both groups received 5 weekly sessions of Tinnitus Retraining Therapy (TRT). Psychoeducational counseling focused on emotional/physical reactions, sound enrichment, and habituation. Delivered per standardized protocol.	TRT/CBT group: Cognitive Behavioral Therapy focusing on cognitive restructuring and coping with tinnitus. TRT/EMDR group: Eye Movement Desensitization and Reprocessing (EMDR) following an 8-phase AIP-based protocol with bilateral stimulation. Both had 5 weekly sessions and one follow-up session.	Both treatments significantly reduced tinnitus distress (TFI) and comorbid symptoms. Over 80% of participants reported subjective improvement. No significant superiority between groups, but distinct trajectories: EMDR led to longer-term gains in some domains. Study supports both TRT/CBT and TRT/EMDR as effective bimodal therapies for tinnitus and comorbid anxiety/depression/hyperacusis.	High

Marks et al., 2023 [40]	102 adults with chronic tinnitus-related insomnia, recruited via advertisement and clinical referrals at a London ENT hospital.	Mini-Tinnitus Questionnaire (Mini-TQ), Tinnitus Questionnaire (TQ), Visual Analogue Scale for tinnitus loudness, Tinnitus Catastrophizing Scale (TCS). Diagnosis confirmed through clinical audiological evaluation.	Depression (PHQ-9), anxiety (GAD-7), psychological distress (CORE-OM). Participants with severe psychiatric illness were excluded. Baseline psychological distress was moderate to high (CORE mean ~11), with depressive and anxious symptoms reported.	CBTi led to significant improvements: ISI (−6.9 to −10.3 points), TQ (−6.1 to −17.4 points), PHQ-9 (−2.2 to −5.4), GAD-7 (up to −3.2), with large effect sizes vs controls. Clinically meaningful improvement in ISI at 6 months: 82% (CBTi) vs 47% (ABC) vs 20% (SSG). CORE-OM: 36% improved in CBTi vs 15% ABC. Improvements in tinnitus and psychiatric symptoms were strongly correlated with sleep improvement.	CBTi program adapted for tinnitus, delivered over 6 sessions in 8 weeks, included sleep restriction, stimulus control, cognitive therapy, relaxation, psychoeducation, and optional sound enrichment.	Psychiatric symptoms (depression, anxiety, distress) were addressed indirectly via CBTi targeting insomnia mechanisms. No separate psychiatric intervention was delivered.	CBTi was significantly more effective than Audiology-Based Care (ABC) and Sleep Support Group (SSG) in reducing insomnia, tinnitus distress, depressive symptoms, and improving functioning and QoL. Unexpectedly, tinnitus loudness also reduced. 82% of CBTi participants had clinically meaningful insomnia reduction. Benefits were maintained at 6 months. CBTi indirectly improved tinnitus via sleep mechanisms and cognitive reframing.	High

McKenna et al., 2017 [41]	75 adults with chronic distressing tinnitus recruited from a UK specialist outpatient clinic.	Tinnitus Questionnaire (TQ), Tinnitus Functional Index (TFI), Visual Analogue Scale (VAS) for loudness, Tinnitus Catastrophizing Scale (TCS), Tinnitus Fear Avoidance Scale (T-FAS), Tinnitus Acceptance Questionnaire (TAQ).	Psychological distress (CORE-NR), anxiety and depression (HADS), avoidance behavior, catastrophizing, reduced mindfulness (MAAS), functional impairment (WSAS). Participants had high distress at baseline (CORE-NR mean = 19.1). Those with severe psychiatric illness were excluded.	MBCT significantly reduced tinnitus distress (TQ mean diff = –6.3 at post; –7.2 at 6-months), with effect sizes up to 0.56. Reliable change in 62% (MBCT) vs 53% (RT). Anxiety, depression, catastrophizing, and avoidance improved. Acceptance and mindfulness gains were significantly greater in MBCT. TFI improvements significant only at 6 months (mean diff = –9.6, *p* = 0.011).	8-week Mindfulness-Based Cognitive Therapy (MBCT) program adapted for tinnitus: weekly 2-hour group sessions. Emphasized sound meditation, tinnitus-specific cognitive models, attentional control, and acceptance.	MBCT targeted emotional distress and maladaptive cognition, though not a standalone psychiatric treatment. Psychological symptoms (e.g., depression/anxiety) were addressed indirectly via mindfulness.	MBCT was more effective than relaxation training (RT) in reducing tinnitus distress, avoidance, catastrophizing, and in increasing acceptance and mindfulness. MBCT benefits persisted and increased over time. Both interventions improved anxiety, depression, and functioning, but MBCT had a broader and longer-lasting effect profile.	High

Moore et al., 2020 [43]	14 adults (7 males, 7 females) from the Norfolk and Norwich University Hospital.	THI	Depression, anxiety, PTSD (after the onset of tinnitus).	Depression: p=0.01 at discharge (T1), and p=0.0054 at 6 months (T6), anxiety : p=0.0625 at discharge (T1), and p=0.3125 at 6 months (T6).	Patients received up to 10 sessions of eye movement desensitization and reprocessing (EMDR) therapy. significant results show that the treatment worked in reducing tinnitus.	N/A	EMDR therapy significantly reduced tinnitus distress post-treatment and at follow-up (THI score). Depression improved significantly post-treatment and at follow-up, anxiety had no significant improvement.	High
Papazian, 2015 [48]	The case of a patient who was analyzed in a psychoanalytic setting.	Tinnitus is discussed as a subjective phenomenon linked to psychological and emotional states. no medical evaluation or standardized tools.	Anxiety, social isolation, depressive traits and challenges in parental relationships, feelings of separation, abandonment and internal conflicts. (after the onset of tinnitus).	N/A	Psychoanalytic therapy to alleviate the psychological distress, even if it doesn't directly eliminate the tinnitus itself.	Psychoanalytic therapy to alleviate the psychological distress, even if it doesn't directly eliminate the tinnitus itself.	Tinnitus is presented as a metaphorical symptom often related to psychological separations or early-life traumas. Psychoanalytic interventions help to understand the symbolic meaning of tinnitus. However, the study doesn't report measurable data.	Low

Park et al., 2020 [23]	9032 adults from the 2010-2012 Korean National Health and Nutrition Examination Survey.	Self-reported.	Stress, depressed mood, suicidal ideation (after the onset of tinnitus).	Stress : $p<0.001$, depressed mood : $p< 0.001$, suicidal ideation : $p<0.001$.	N/A	N/A	Tinnitus was significantly associated with stress, depressed mood and suicidal ideation.	Moderate
Park et al., 2023 [26]	82 patients (28 males, 54 females) from a tinnitus clinic in South Korea.	THI	Anxiety, depression, moderate stress (before tinnitus).	Psychiatric symptoms and tinnitus distress : $p=0.004$.	N/A	Recommended, but not described in this study.	The presence of psychiatric symptoms independently increases tinnitus-related distress.	Moderate High

Sahlsten et al., 2017 [29]	83 patients with chronic, non-pulsatile tinnitus from two hospitals in Finland.	Diagnosis : detailed clinical interview and audiological evaluations conducted by ear, nose, and throat specialists., evaluation : THI and visual analogue scale (vas).	Major depression, dysthymia, panic disorder, agoraphobia, alcohol dependence, dissociative disorders, obsessive-compulsive personality disorder (OCPD), avoidant personality disorder, schizoid personality disorder (before and after tinnitus).	Major depression associated with tinnitus intensity: p=0.27, major depression associated with tinnitus annoyance : p=0.30, major depression associated with THI grade : p=0.30, dysthymia (not mentioned), panic disorder (not mentioned), agoraphobia (not mentioned), alcohol dependence (not mentioned), dissociative disorders associated with tinnitus intensity : p=0.34, dissociative disorders associates with tinnitus	N/A	N/A	Tinnitus was not significantly associated with axis i or axis ii disorders based on the p-values. findings suggest that psychiatric disorders in tinnitus patients may reflect shared etiological factors (e. g., brain dopamine dysregulation).	High

				annoyance : p=0.34, dissociative disorders associated with THI score : p=0.34, OCPD associated with tinnitus intensity : p=0.43, OCPD associated with tinnitus annoyance : p=0.43, OCPD associated with THI score : p=0.43, avoidant personality disorder (not mentioned), schizoid personality disorder (not mentioned).					

Salviati et al., 2014 [49]	239 patients (151 males, 88 females) from Rome.	Diagnosed using THI and scl-90-r screening tools.	Somatization, obsessive-compulsive personality disorder, sensitivity, depression, anxiety, hostility, phobic anxiety, paranoid ideation, psychosis (after the onset of tinnitus).	p<0.001 between tinnitus and somatization, obsessive-compulsive personality disorder, sensitivity, depression, anxiety, hostility, phobic anxiety, paranoid ideation, psychosis.	N/A	Psychotropic drugs (not evaluated).	Significant correlation between tinnitus and psychiatric comorbidities.	Moderate
Wang et al., 2021 [33]	296 patients with chronic tinnitus.	THI	Self-esteem issues, resilience issues, poor social support (before the onset of tinnitus).	Significant negative correlation between resilience and tinnitus distress (p = 0, 001), significant negative correlation between social support and tinnitus distress (p < 0, 001), significant negative correlation between	N/A	Building resilience and self-esteem.	Social support indirectly affects tinnitus distress via self-esteem and resilience.	High

				self-esteem and tinnitus distress (p < 0, 001).				
Weise et al., 2016 [39]	124 adult participants with chronic tinnitus-related distress in Germany, recruited via online and public media.	THI, Mini-Tinnitus Questionnaire (Mini-TQ), self-reported loudness, hearing impairment, number of past treatments. Diagnosed tinnitus confirmed by ENT specialist.	Anxiety, depression, insomnia (measured by HADS and ISI), and tinnitus-related acceptance/ experiential avoidance (measured by the Tinnitus Acceptance Questionnaire). Severe psychiatric disorders were excluded through diagnostic interview and web screening.	Large effect sizes for reduction in tinnitus distress (THI: g = 0.83; Mini-TQ: g = 1.08) and medium to small effects for psychiatric symptoms (e.g., HADS anxiety: g = 0.35; HADS depression: g = 0.36; ISI: g = 0.66). Reliable change achieved in 72.6% (THI) and 80.6% (Mini-TQ) of iCBT participants post-treatment.	10-week Internet-based CBT program with 12 mandatory and 6 optional modules including relaxation, cognitive restructuring, exposure, sleep, hearing, and concentration modules. Weekly therapist guidance via secure online messaging.	10-week Internet-based CBT program with 12 mandatory and 6 optional modules including relaxation, cognitive restructuring, exposure, sleep, hearing, and concentration modules. Weekly therapist guidance via secure online messaging.	iCBT led to significantly greater reductions in tinnitus-related distress, anxiety, depression, and insomnia compared to an online discussion forum. Improvements were sustained at 6-month and 1-year follow-ups. High rates of reliable change and functional improvement. Sleep gains slightly diminished over time.	High

Xu et al., 2016 [32]	543 patients from an ENT clinic in China.	THI	Anxiety, depression (after the onset of tinnitus).	p=0.000 between tinnitus and anxiety, p=0.000 between tinnitus and depression.	N/A	N/A	Anxiety and depression are strongly associated with tinnitus severity.	Moderate

Acronyms: CBT: Cognitive Behavioral Therapy, CMDQ: Common Mental Disorders Questionnaire, EMDR: Eye Movement Desensitization and Reprocessing, ENT: Ear, Nose, and Throat, GIN: Gaps-in-Noise, GWAS: Genome-Wide Association Study, HADS: Hospital Anxiety and Depression Scale, JBI: Joanna Briggs Institute, MMAT: Mixed Methods Appraisal Tool, MRI: Magnetic Resonance Imaging, OCPD: Obsessive-Compulsive Personality Disorder, PTSD: Post-Traumatic Stress Disorder, RCT: Randomized Controlled Trial, SAS: Self-Rating Anxiety Scale, SCL-90: Symptom Checklist-90, SDS: Self-Rating Depression Scale, SNP: Single Nucleotide Polymorphism, SSRI: Selective Serotonin Reuptake Inhibitor, TEOAE: Transient-Evoked Otoacoustic Emissions, TFI: Tinnitus Functional Index, THI: Tinnitus Handicap Inventory, VAS: Visual Analogue Scale
